# CHS-DRG 2.0 and insurance-specific patient cost sharing in gestational diabetes admissions: a regression discontinuity in time study

**DOI:** 10.3389/fpubh.2026.1939491

**Published:** 2026-09-01

**Authors:** Senhua Chen, Shen Huang, Ling Lan, Yulong Zhang

**Affiliations:** Fujian Maternity and Child Health Hospital, Fuzhou, China

**Keywords:** CHS-DRG 2.0, gestational diabetes mellitus, health insurance, patient cost sharing, regression discontinuity in time

## Abstract

**Objectives:**

Diagnosis-related group reform aims to restrain inpatient spending, but obstetric evaluations commonly emphasize average hospital costs rather than insurance-specific patient cost sharing. We evaluated early discontinuities associated with CHS-DRG 2.0 in hospitalization costs, out-of-pocket (OOP) payment, self-pay ratio, and selected perinatal outcomes among gestational diabetes mellitus (GDM) admissions.

**Methods:**

We analyzed 8,690 singleton GDM delivery admissions at a tertiary maternal and child health hospital in Fujian, China. The main cohort excluded eight stays exceeding 60 days, consistent with the policy DRG-management scope. We classified policy period from the recorded admission date under the provincial rule: admissions before 1 January 2025, including cross-cutoff stays, remained under the prior payment arrangement; admissions on or after that date entered the CHS-DRG 2.0 policy period. Actual DRG assignment and claim settlement were unavailable. Local linear regression discontinuity in time models used triangular kernels, data-driven bandwidths, robust bias-corrected confidence intervals, and discharge-day-clustered standard errors.

**Results:**

The admission-date cutoff was not associated with a precise discontinuity in total hospitalization cost (estimate, −42.10 CNY; 95% CI, −1,158.46 to 1,074.26; *p* = 0.941), OOP payment (−102.15 CNY; 95% CI, −1,289.55 to 1,085.25; *p* = 0.866), or overall self-pay ratio (−2.75 percentage points; 95% CI, −8.06 to 2.56; *p* = 0.310). The self-pay ratio declined among resident-insured admissions (−12.41 percentage points; 95% CI, −20.05 to −4.77; *p* = 0.001), but not employee-insured admissions (−0.33 percentage points; 95% CI, −5.79 to 5.13; *p* = 0.905). The interaction estimate was −11.99 percentage points (95% CI, −19.85 to −4.13; *p* = 0.003). No exploratory perinatal outcome remained significant after false-discovery-rate correction.

**Conclusion:**

The admission-date policy boundary was associated with a larger reduction in relative patient cost sharing among resident-insured GDM admissions. Discontinuous admission density at the cutoff and possible concurrent calendar-time changes limit causal attribution and preclude inference about a specific payment mechanism. Payment-reform evaluations should jointly assess insurance-specific patient cost sharing, provider expenditure, and clinical outcomes.

## 1 Introduction

Diagnosis-related group (DRG) payment is a major instrument for containing inpatient expenditure and reducing unwarranted variation in hospital resource use in China (1, 2). In Fujian Province, a province-wide transition notice required covered public hospitals to fully switch to the national DRG 2.0 version from 1 January 2025. CHS-DRG 2.0 further standardized grouping and payment rules, but its early effects may differ across clinical specialties and insurance arrangements. Obstetric care is a particularly relevant setting because payment incentives must operate within time-sensitive maternal and neonatal safety requirements. International experience also frames DRG systems as tools for transparency and efficiency that require continuing attention to quality and distributional consequences (3).

Gestational diabetes mellitus (GDM) is common in China and is associated with maternal and neonatal morbidity (4, 5). It also creates substantial short-term costs for hospitals and households (6). These clinical and economic features make GDM delivery admissions relevant for evaluating whether changes at a payment-policy boundary coincide with patient-facing cost sharing without clear evidence of deterioration in selected perinatal outcomes.

Most evaluations of payment reform report average cost, length of stay, service efficiency, or aggregate quality outcomes (7–9). Average effects can conceal distributional changes when benefit structures determine how hospital charges are divided between insurance funds and patients. Childbirth-related financial burden varies by insurance coverage (10), yet obstetric DRG studies rarely assess whether relative patient cost sharing changes differently under employee and resident medical insurance.

We therefore used an admission-date regression discontinuity in time (RDiT) design to assess early discontinuities associated with CHS-DRG 2.0 in hospitalization cost, out-of-pocket (OOP) payment, self-pay ratio, and selected perinatal indicators among GDM admissions. We also tested whether the discontinuity in self-pay ratio differed between employee and resident medical insurance. The study extends provider-centered payment evaluation by examining relative patient cost sharing and insurance-related distributional effects.

## 2 Materials and methods

### 2.1 Study design and setting

This retrospective observational study evaluated an admission-date discontinuity at 1 January 2025. The provincial notice implementing the national and Fujian 2.0 grouping policies listed Fujian Maternity and Child Health Hospital among 14 covered provincial institutions, applied DRG management to insured stays of 60 days or fewer, and specified that patients admitted before 1 January 2025 but not yet discharged would remain under the previous payment policy. RDiT estimates a discontinuity at an intervention date while allowing separate local time trends on either side of the cutoff (11, 12).

### 2.2 Data source and study population

The study was conducted at Fujian Maternity and Child Health Hospital, a tertiary maternal and child health hospital in Fuzhou, China. Data were extracted from routinely maintained electronic hospitalization and delivery-registration records for admissions from 1 January 2024 through 31 December 2025.

Eligible admissions were patients with GDM identified from a principal or secondary discharge diagnosis coded ICD-10 O24.4-O24.9 and with exactly one infant recorded in the inpatient delivery registry. In accordance with the provincial policy defining the scope of DRG management, we excluded admissions with a length of stay exceeding 60 days. Total hospitalization cost, OOP payment, admission date, discharge date, and length of stay were complete; therefore, no records were excluded for missing core variables. The final analytic cohort comprised 8,690 admissions: 4,658 before and 4,032 on or after the admission-date cutoff. After admission dates were retrieved, 56 admissions were identified as spanning the cutoff; two had stays exceeding 60 days and were excluded, leaving 54 eligible cross-cutoff admissions. These 54 admissions were classified in the pre-policy group according to their admission date, as required by the policy.

### 2.3 Outcomes and covariates

The prespecified primary outcome was total hospitalization cost per admission. Secondary economic outcomes were OOP payment, the self-pay ratio, and length of stay. The self-pay ratio was calculated as OOP payment divided by total hospitalization cost and expressed as a percentage. Ten cost components were examined as exploratory outcomes: medical service, therapeutic procedure, delivery and surgery, nursing, laboratory and pathology, imaging, clinical diagnostic project, drug, blood product, and consumable fees. All monetary outcomes were expressed in Chinese yuan (CNY).

Insurance was categorized as employee medical insurance, resident medical insurance, self-pay, or other insurance or assistance. In the source data, these categories were coded as 0, 1, 2, and 3, respectively, and were treated as nominal rather than ordered. Heterogeneity analysis was restricted to employee- and resident-insured admissions. Delivery type was coded for balance testing as a binary indicator (1 = caesarean delivery; 0 = other delivery modes; source code 2 versus codes 0/1). Baseline covariates were age, gestational age at delivery, gravidity, parity, delivery type, and insurance category.

Exploratory perinatal outcomes were neonatal asphyxia, birth trauma, and postpartum haemorrhage. Neonatal asphyxia was defined as a 1-min or 5-min Apgar score of 7 or lower in the delivery registry (13, 14). Birth trauma was identified from discharge diagnoses coded as ICD-10 O70.2 or O70.3-O70.9. Postpartum haemorrhage was defined as blood loss of at least 500 mL within 24 h after fetal delivery. Birth trauma and postpartum haemorrhage were analyzed separately (15).

### 2.4 Statistical analysis

Continuous variables are reported as median (25th percentile, 75th percentile), and categorical variables as counts and percentages. Descriptive policy-period comparisons used Wilcoxon rank-sum tests for continuous outcomes and Pearson chi-square or Fisher exact tests for categorical outcomes.

The running variable was the number of calendar days between the recorded admission date and 1 January 2025. Local linear RDiT models used triangular kernels and mean-squared-error-optimal bandwidths. We reported robust bias-corrected estimates, 95% confidence intervals (CIs), and two-sided *p* values (16). Standard errors were clustered by discharge day to account for within-day correlation. Binary outcomes were analyzed as local linear probability models and reported as risk differences in percentage points. Estimates, robust standard errors, and confidence intervals for binary outcomes were converted from the proportion scale to percentage points by multiplying by 100. Because the running variable was discrete in days, mass-point adjustment was enabled in all RD models.

The primary analysis estimated discontinuities in total cost, OOP payment, self-pay ratio, and length of stay. Ten cost components and three perinatal outcomes were exploratory. Benjamini-Hochberg correction was applied separately to the cost-component and perinatal-outcome families; nominal *p* values and false-discovery-rate-adjusted *q* values were retained. No FDR correction was applied to the prespecified economic outcomes.

Insurance heterogeneity was assessed with separate RDiT models for employee- and resident-insured admissions and a pooled local linear model with policy, resident-insurance status, running time, and all interactions. The policy-by-resident coefficient was the prespecified heterogeneity test. Sensitivity analyses included covariate balance tests, an admission-density test, pre-policy placebo cutoffs, bandwidth multipliers, local quadratic specifications, exclusion of cross-cutoff stays, and inclusion of stays exceeding 60 days. Analyses used R version 4.6.1 with rdrobust 4.0.0 and rddensity 3.0; readxl, dplyr, sandwich, lmtest, ggplot2, and jsonlite supported data processing, clustered inference, visualization, and reporting.

### 2.5 Ethics, consent, and permissions

The Ethics Committee of Fujian Maternity and Child Health Hospital approved the study (2026KY243-01). The requirement for individual informed consent was waived because the study used retrospective, de-identified hospitalization data.

## 3 Results

### 3.1 Cohort characteristics

The main cohort included 8,690 GDM delivery admissions, with 4,658 before and 4,032 after the admission-date cutoff. Median age and gestational age at delivery were 32 years and 39 weeks in both periods. Employee medical insurance covered 70.8% of pre-policy and 70.4% of post-policy admissions; resident medical insurance covered 21.9 and 19.9%, respectively (Table 1).

**Table 1:** Characteristics of eligible GDM delivery admissions before and after the admission-date CHS-DRG 2.0 cutoff.

Characteristic	Pre-policy	Post-policy
Age, years	32.00 (29.00, 35.00)	32.00 (30.00, 35.00)
Gestational age at delivery, weeks	39.00 (38.00, 39.00)	39.00 (38.00, 39.00)
Gravidity	2.00 (1.00, 3.00)	2.00 (1.00, 3.00)
Parity	1.00 (1.00, 2.00)	1.00 (1.00, 2.00)
Cesarean delivery	2,165 (46.5%)	1,812 (44.9%)
Employee medical insurance	3,299 (70.8%)	2,840 (70.4%)
Resident medical insurance	1,020 (21.9%)	804 (19.9%)
Self-pay	221 (4.7%)	282 (7.0%)
Other insurance or assistance	118 (2.5%)	106 (2.6%)

Continuous variables are reported as median (IQR), and categorical variables as n (%).

### 3.2 Economic outcomes and perinatal indicators

**Figure 1:**
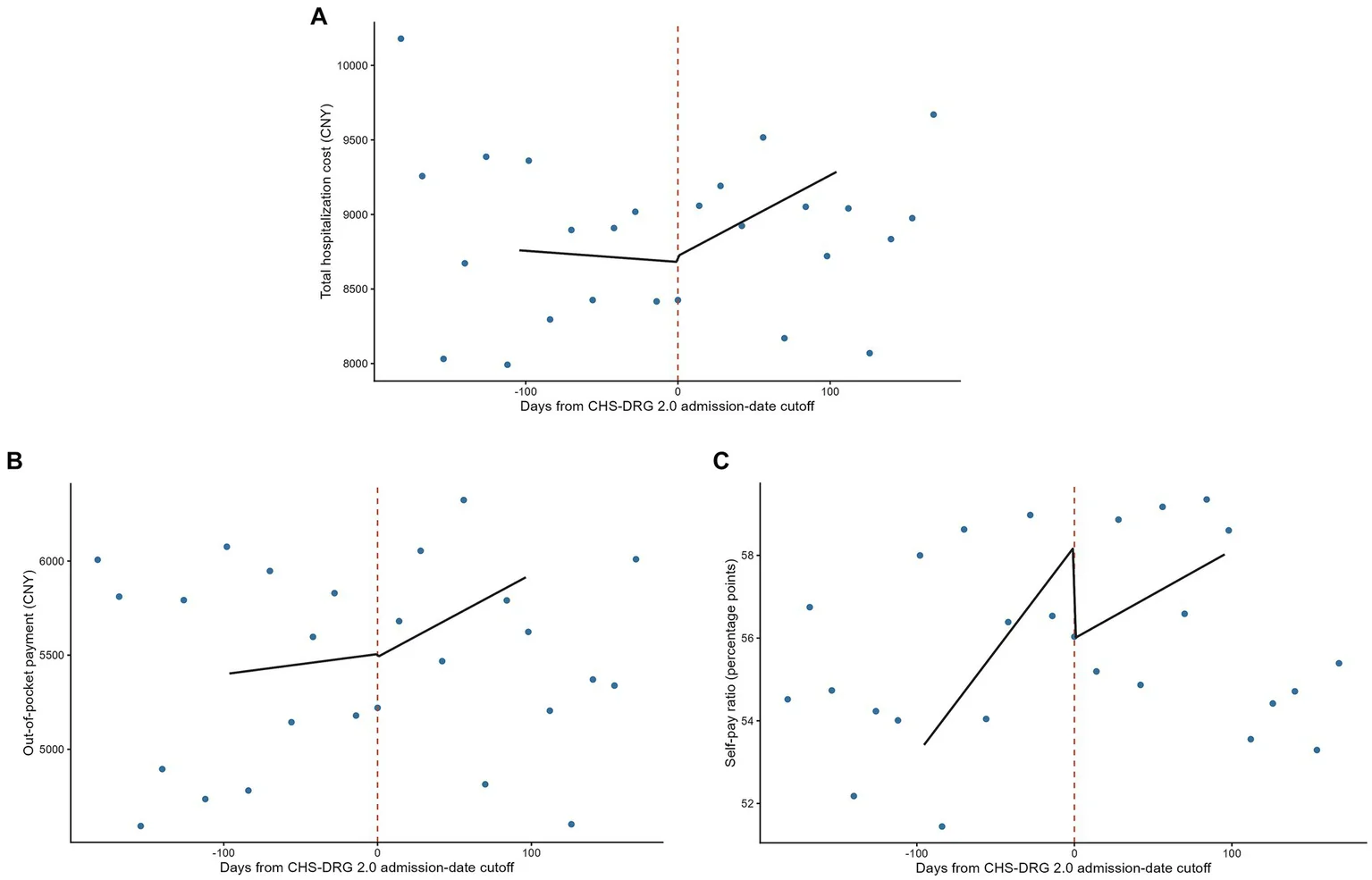
Regression discontinuity in time plots for economic outcomes using the admission-date cutoff. **(A)** Total hospitalization cost. **(B)** OOP payment. **(C)** Self-pay ratio. Points show 14-day binned means within 180 days of 1 January 2025. Lines show triangular-kernel local linear fits within the outcome-specific bandwidth. The dashed line marks the cutoff.

Descriptive medians were lower after the cutoff for total cost, OOP payment, and self-pay ratio, but these comparisons did not account for time trends (Supplementary Table S1). The primary RDiT model did not detect a precise discontinuity in total hospitalization cost (estimate, −42.10 CNY; 95% CI, −1,158.46 to 1,074.26; *p* = 0.941), OOP payment (−102.15 CNY; 95% CI, −1,289.55 to 1,085.25; *p* = 0.866), self-pay ratio (−2.75 percentage points; 95% CI, −8.06 to 2.56; *p* = 0.310), or length of stay (−0.08 days; 95% CI, −0.62 to 0.45; *p* = 0.759) (Table 2; Figure 1).

**Table 2:** Main economic and exploratory perinatal RDiT estimates.

Outcome	Estimate	Robust SE	*p* value	95% CI lower	95% CI upper	Effective sample size	*q* value
Total hospitalization cost, CNY	−42.102	569.58	0.941	−1,158.46	1,074.26	2,653	NA
Out-of-pocket payment, CNY	−102.15	605.83	0.866	−1,289.55	1,085.25	2,448	NA
Self-pay ratio, percentage points	−2.751	2.711	0.310	−8.064	2.563	2,425	NA
Length of stay, days	−0.084	0.273	0.759	−0.620	0.452	3,314	NA
Neonatal asphyxia, risk difference (percentage points)	−0.69	0.64	0.274	−1.94	0.55	3,248	0.823
Birth trauma, risk difference (percentage points)	−0.37	0.70	0.598	−1.74	1.00	2,621	0.851
Postpartum haemorrhage, risk difference (percentage points)	0.22	1.18	0.851	−2.09	2.54	2,742	0.851

*q* values are not applicable (NA) for prespecified economic outcomes. Benjamini-Hochberg false-discovery-rate correction was applied only within the exploratory cost-component and perinatal-outcome families.

No exploratory perinatal outcome showed a statistically significant discontinuity after FDR correction. The estimates were −0.69 percentage points for neonatal asphyxia (*q* = 0.823), −0.37 percentage points for birth trauma (*q* = 0.851), and 0.22 percentage points for postpartum haemorrhage (*q* = 0.851) (Table 2).

### 3.3 Insurance heterogeneity

**Figure 2:**
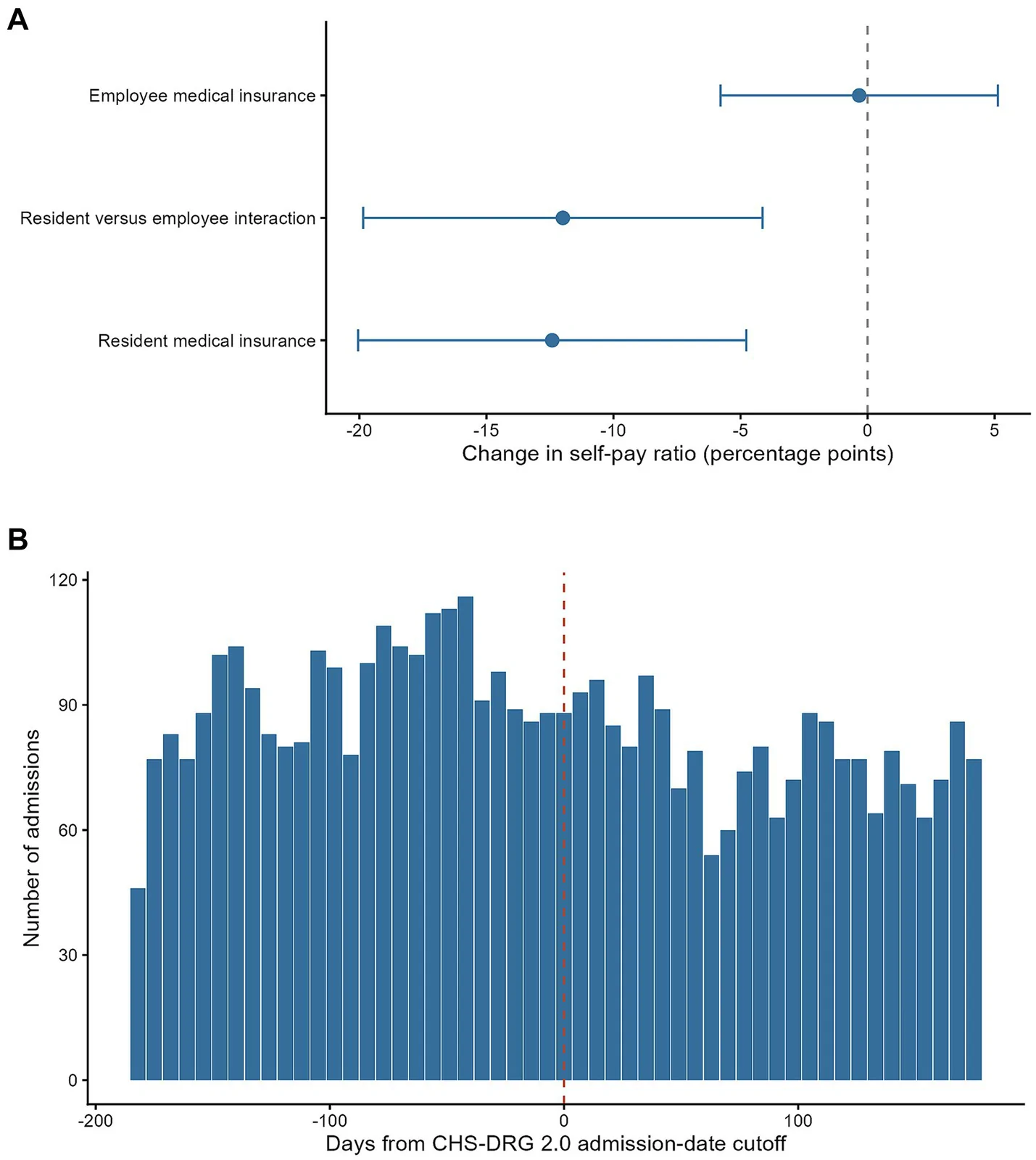
Insurance-specific discontinuities in the self-pay ratio. Points and horizontal lines show robust bias-corrected estimates and 95% confidence intervals for employee-insured admissions, resident-insured admissions, and the resident-versus-employee interaction.

Insurance-specific policy-period characteristics are reported in Supplementary Table S2. Neither total hospitalization cost nor absolute OOP payment showed a precise discontinuity in either insurance subgroup (Supplementary Table S5). The employee-insured subgroup showed no discontinuity in self-pay ratio (estimate, −0.33 percentage points; 95% CI, −5.79 to 5.13; *p* = 0.905). The resident-insured subgroup showed a reduction of 12.41 percentage points (95% CI, −20.05 to −4.77; *p* = 0.001). The pooled resident-versus-employee interaction was −11.99 percentage points (95% CI, −19.85 to −4.13; *p* = 0.003) (Table 3; Figure 2).

**Table 3:** Insurance-specific RDiT estimates for the self-pay ratio.

Outcome	Estimate	Robust SE	p value	95% CI lower	95% CI upper	Effective sample size
Employee medical insurance	−0.331	2.785	0.905	−5.789	5.127	1,632
Resident medical insurance	−12.413	3.898	0.001	−20.053	−4.773	637
Interaction: resident versus employee	−11.992	4.009	0.003	−19.849	−4.135	2,130

### 3.4 Validity and sensitivity analyses

RD-based balance tests did not identify statistically significant discontinuities in continuous covariates, the binary delivery-type indicator, or the four insurance-category indicators after multiplicity correction (Supplementary Table S6). Pre-policy placebo cutoff estimates for total hospitalization cost at 1 April and 1 July 2024 were not statistically significant (*p* = 0.823 and *p* = 0.443, respectively; Supplementary Table S7). The total-cost estimate remained imprecise across bandwidth, local quadratic, cross-cutoff-exclusion, and long-stay-inclusion analyses (Supplementary Tables S8 and S9). The resident-insurance self-pay-ratio estimate was sensitive to bandwidth selection: at 0.5 times the data-driven bandwidth, the estimate was −0.43 percentage points (95% CI, −11.61 to 10.75; *p* = 0.940), whereas it remained negative at the main, 1.5-times, and 2-times bandwidths (−12.41, −11.59, and −14.00 percentage points; *p* = 0.001, 0.003, and <0.001, respectively; Supplementary Table S8). It also remained negative when cross-cutoff stays were excluded (−17.96 percentage points; 95% CI, −26.97 to −8.95; *p* < 0.001; Supplementary Table S9). The resident-versus-employee interaction estimate showed the same qualitative pattern: it was imprecise at 0.5 times the data-driven bandwidth (−7.09 percentage points; 95% CI, −17.14 to 2.95; *p* = 0.166) and remained negative at the main, 1.5-times, and 2-times bandwidths (−11.99, −13.49, and −15.09 percentage points; *p* = 0.003, <0.001, and <0.001, respectively; Supplementary Table S8).

The local admission-density test indicated a discontinuity at the cutoff (jackknife *p* = 0.004; Supplementary Table S10; Supplementary Figure S1). This diagnostic does not support an assumption of smooth admission density and reinforces the need for cautious, non-causal interpretation of the RDiT estimates.

## 4 Discussion

In this admission-date analysis, the main detectable temporal discontinuity was a reduction in the self-pay ratio among resident-insured admissions relative to employee-insured admissions. We detected no precise discontinuities in total hospitalization cost, absolute OOP payment, the overall self-pay ratio, or length of stay. By contrast, the self-pay ratio declined among resident-insured admissions but not among employee-insured admissions. The pooled interaction further indicated that the change differed between the two insurance groups. Because the self-pay ratio is calculated as OOP payment divided by total hospitalization cost, this finding concerns relative patient cost sharing. It does not establish a reduction in absolute OOP payment or an improvement in household financial protection.

The exploratory perinatal analyses did not identify any discontinuity that remained statistically significant after false-discovery-rate correction. The selected indicators therefore did not reveal a clear adverse temporal change. However, they do not establish preserved or improved maternal or neonatal safety. The small number of events and imprecision of local estimates warrant cautious interpretation.

The absence of a precise average-cost discontinuity is consistent with mixed evidence from Chinese DRG evaluations. Estimated effects vary by hospital level, specialty, implementation phase, and analytic design (9, 12, 17–19). European experience similarly frames DRG systems as instruments for improving transparency, efficiency, and quality, while emphasizing the importance of payment architecture and institutional context (3). Evidence from hospital-system reforms outside China further supports evaluating performance beyond expenditure alone, including access, service coordination, and quality or resilience (20, 21). These studies are not direct evaluations of CHS-DRG payment and cannot establish the effect of CHS-DRG 2.0. Obstetric services may have limited short-term flexibility because delivery care, staffing, and maternal-neonatal safety capacity require substantial fixed and standby resources (22).

CHS-DRG 2.0 was an upgrade of the pre-existing DRG payment arrangement rather than a transition from fee-for-service alone. The policy notice specified 774 DRG groups paid by the DRG method, 50 complex groups temporarily paid by item, and 15 mental-health groups paid by bed-day; payment standards were based on the base rate, DRG weight, and hospital coefficient (23). Obstetric delivery-related groups are not named among the listed item-paid or bed-day exceptions. Thus, eligible GDM delivery admissions had policy eligibility for DRG management, but our data do not identify the actual DRG group, payment standard, or settlement pathway for any admission. In addition, review of applicable policy documents and hospital administrative records did not identify concurrent changes in benefit policies, charge schedules, coding practices, clinical pathway policies, or hospital management at the cutoff. This review did not identify an observed concurrent change, but cannot exclude unobserved contemporaneous changes and does not identify a patient-level payment or settlement mechanism.

Nevertheless, the mechanism underlying the resident-insurance finding remains uncertain. We lacked admission-level data on DRG assignment, claim adjudication, settlement, insurance-fund payment, service-level reimbursement status, prescribing, household income, and supplemental coverage. We therefore could not determine whether the observed change reflected service composition, provider behaviour, or other unmeasured administrative processes. Insurance-fund payment was not derived as total hospitalization cost minus OOP payment because other payment sources may have contributed to the recorded total.

This study has several strengths, including patient-level accounting data, recorded admission dates, an explicit policy-rule-based classification of cross-cutoff stays, insurance-specific analyses, date-clustered inference, multiplicity correction, and multiple sensitivity analyses. However, the study was conducted at a single tertiary maternal and child health hospital. Its findings may not generalize to other regions or provider types. Measured continuous covariates and the binary delivery-type indicator showed no statistically significant discontinuities after correction, and placebo estimates were not statistically significant. The four binary insurance-category indicators also showed no statistically significant discontinuities after multiplicity correction. However, the admission-density diagnostic was discontinuous at the cutoff (jackknife *p* = 0.004). Together with the absence of an untreated comparison series, this finding requires cautious causal interpretation. The resident-insurance estimate was near zero and imprecise at the narrowest bandwidth, despite remaining negative at the main and wider bandwidths; the resident-versus-employee interaction was imprecise at the narrowest bandwidth but remained negative at the main and wider bandwidths. Discrete daily running time and date clustering may also affect asymptotic approximations. Rare perinatal outcomes further limited precision.

Future multicentre studies should combine longer follow-up with patient-level linkage of DRG assignment, claim adjudication and settlement records, insurance-fund payment amounts, service-level reimbursement status, prescribing data, and supplemental coverage. Such designs could more credibly distinguish policy timing, provider responses, changes in patient payment, and the mechanisms underlying insurance-specific heterogeneity.

## 5 Conclusion

Among eligible singleton GDM delivery admissions in one hospital, the CHS-DRG 2.0 admission-date boundary was not associated with a precise discontinuity in average hospitalization cost, absolute OOP payment, or overall self-pay ratio. A larger reduction in self-pay ratio was observed among resident-insured than employee-insured admissions. Because admission density was discontinuous and concurrent changes cannot be excluded, our estimates should therefore not be interpreted as evidence of a specific causal mechanism. Payment-reform evaluations should examine insurance-specific patient cost sharing, provider expenditure, and clinical outcomes together.

## Data Availability

The original contributions presented in the study are included in the article/Supplementary material, further inquiries can be directed to the corresponding author.
