# Supplementary material for: CHS-DRG 2.0 and insurance-specific patient cost sharing in gestational diabetes admissions: a regression discontinuity in time study
Source: Front Public Health. 2026 Sep 1;14:1939491. doi: 10.3389/fpubh.2026.1939491 (PMC13575385; doi:10.3389/fpubh.2026.1939491)
Supplement: Supplementary file 1 [file Data_Sheet_1.docx]

Supplementary Material

# 1 Supplementary Methods

Supplementary Methods note: RDiT estimates are robust bias-corrected local linear estimates. Robust SE denotes the robust standard error, and effective sample size denotes observations within the selected bandwidth. Bandwidths were selected by the mean-squared-error-optimal procedure; mass-point adjustment accounts for the discrete daily running variable. q values are Benjamini-Hochberg false-discovery-rate-adjusted p values and apply only to the stated exploratory outcome families. Descriptive pre-policy versus post-policy comparisons use Wilcoxon rank-sum tests for continuous variables and Pearson chi-square or Fisher exact tests for categorical variables, as identified in each table. Tables S1 and S4 are unadjusted descriptive comparisons; they do not account for time trends and are not interpreted as policy effects. Comparable bandwidth-sensitivity analyses were performed for both the resident-insurance estimate and the resident-versus-employee interaction estimate.

# 2 Supplementary Data

This file contains descriptive, exploratory, design-validity, and sensitivity analyses supporting the main manuscript.

# 3 Supplementary Figures and Tables

## 3.1 Supplementary Tables

Table S1. Descriptive economic outcomes before and after the admission-date cutoff.

| **Outcome** | **Pre-policy median (IQR)** | **Post-policy median (IQR)** | **Statistical test** | **p value** |
| --- | --- | --- | --- | --- |
| Total hospitalization cost, CNY | 7534.77 (6125.83, 9490.47) | 7435.95 (5944.82, 9382.91) | Wilcoxon rank-sum test | 0.013 |
| Out-of-pocket payment, CNY | 4278.05 (2410.18, 6293.20) | 3833.91 (2332.94, 5774.14) | Wilcoxon rank-sum test | <0.001 |
| Self-pay ratio, percentage points | 57.75 (37.94, 76.59) | 54.33 (39.25, 70.06) | Wilcoxon rank-sum test | <0.001 |
| Length of stay, days | 4.00 (3.00, 5.00) | 4.00 (3.00, 5.00) | Wilcoxon rank-sum test | 0.155 |

Note: This unadjusted pre-policy versus post-policy comparison does not account for time trends and is not interpreted as a policy effect.

Table S2. Descriptive characteristics by insurance scheme and policy period.

| **Characteristic** | **Employee pre-policy** | **Employee post-policy** | **Resident pre-policy** | **Resident post-policy** |
| --- | --- | --- | --- | --- |
| Admissions, n | 3,299 | 2,840 | 1,020 | 804 |
| Age, years | 32.00 (29.00, 35.00) | 32.00 (30.00, 35.00) | 32.00 (29.00, 35.00) | 32.00 (29.00, 36.00) |
| Gestational age at delivery, weeks | 39.00 (38.00, 39.00) | 39.00 (38.00, 39.00) | 39.00 (38.00, 39.00) | 38.00 (37.75, 39.00) |
| Gravidity | 2.00 (1.00, 3.00) | 2.00 (1.00, 3.00) | 2.00 (1.00, 3.00) | 2.00 (1.00, 4.00) |
| Parity | 1.00 (1.00, 2.00) | 1.00 (1.00, 2.00) | 2.00 (1.00, 2.00) | 2.00 (1.00, 2.00) |
| Cesarean delivery | 1,488 (45.1%) | 1,202 (42.3%) | 516 (50.6%) | 421 (52.4%) |

*Table S2 note: Values are n or median (IQR) unless otherwise indicated. Cesarean delivery is n (%). These descriptive comparisons do not account for time trends and are not interpreted as policy effects.*

Table S3. Exploratory cost-component RDiT estimates with false-discovery-rate correction.

| **Outcome** | **Estimate** | **Robust SE** | **p value** | **95% CI lower** | **95% CI upper** | **Effective sample size** | **q value** |
| --- | --- | --- | --- | --- | --- | --- | --- |
| Medical service fees, CNY | 6.756 | 485.84 | 0.989 | -945.47 | 958.98 | 2,319 | 0.989 |
| Therapeutic procedure fees, CNY | 6.423 | 24.334 | 0.792 | -41.271 | 54.116 | 1,231 | 0.989 |
| Delivery and surgical treatment fees, CNY | -176.99 | 154.32 | 0.251 | -479.44 | 125.47 | 1,812 | 0.989 |
| Nursing fees, CNY | 7.412 | 19.054 | 0.697 | -29.933 | 44.757 | 2,130 | 0.989 |
| Laboratory and pathology diagnostic fees, CNY | 35.704 | 47.922 | 0.456 | -58.221 | 129.63 | 2,537 | 0.989 |
| Imaging examination fees, CNY | -45.949 | 40.026 | 0.251 | -124.40 | 32.501 | 2,509 | 0.989 |
| Clinical diagnostic project fees, CNY | -10.447 | 35.469 | 0.768 | -79.965 | 59.071 | 2,621 | 0.989 |
| Drug fees, CNY | 37.194 | 49.129 | 0.449 | -59.097 | 133.49 | 1,983 | 0.989 |
| Blood and blood product fees, CNY | -9.419 | 70.795 | 0.894 | -148.17 | 129.34 | 3,115 | 0.989 |
| Consumables fees, CNY | -13.000 | 36.208 | 0.720 | -83.967 | 57.966 | 3,530 | 0.989 |

Table S4. Descriptive perinatal outcomes before and after the admission-date cutoff.

| **Outcome** | **Pre-policy events** | **Pre-policy total** | **Post-policy events** | **Post-policy total** | **Statistical test** | **p value** |
| --- | --- | --- | --- | --- | --- | --- |
| Neonatal asphyxia | 45 | 4,658 | 32 | 4,032 | Pearson chi-square test | 0.392 |
| Birth trauma | 37 | 4,658 | 34 | 4,032 | Pearson chi-square test | 0.801 |
| Postpartum haemorrhage | 81 | 4,658 | 106 | 4,032 | Pearson chi-square test | 0.004 |

Note: This unadjusted pre-policy versus post-policy comparison does not account for time trends and is not interpreted as a policy effect.

Table S5. Insurance-specific economic RDiT estimates.

| **Insurance group** | **Outcome** | **Estimate** | **Robust SE** | **p value** | **95% CI lower** | **95% CI upper** | **Effective sample size** |
| --- | --- | --- | --- | --- | --- | --- | --- |
| Employee medical insurance | Total hospitalization cost, CNY | -103.67 | 457.79 | 0.821 | -1,000.92 | 793.59 | 1,350 |
| Resident medical insurance | Total hospitalization cost, CNY | -150.69 | 758.71 | 0.843 | -1,637.73 | 1,336.35 | 645 |
| Employee medical insurance | Out-of-pocket payment, CNY | 173.60 | 396.58 | 0.662 | -603.68 | 950.88 | 1,482 |
| Resident medical insurance | Out-of-pocket payment, CNY | -899.51 | 625.90 | 0.151 | -2,126.24 | 327.23 | 602 |
| Employee medical insurance | Self-pay ratio, percentage points | -0.331 | 2.785 | 0.905 | -5.789 | 5.127 | 1,632 |
| Resident medical insurance | Self-pay ratio, percentage points | -12.413 | 3.898 | 0.001 | -20.053 | -4.773 | 637 |

*Table S5 note: Insurance-fund payment, DRG assignment, claim adjudication, and settlement fields were unavailable. Fund payment was not inferred as total cost minus OOP payment because other payment sources may exist.*

Table S6. RD-based covariate balance tests.

| **Outcome** | **Estimate** | **Robust SE** | **p value** | **95% CI lower** | **95% CI upper** | **Effective sample size** | **q value** |
| --- | --- | --- | --- | --- | --- | --- | --- |
| Age, years | 0.237 | 0.357 | 0.507 | -0.463 | 0.938 | 3,599 | 0.633 |
| Gestational age at delivery, weeks | -0.032 | 0.165 | 0.847 | -0.354 | 0.291 | 2,653 | 0.847 |
| Gravidity | -0.099 | 0.135 | 0.464 | -0.364 | 0.166 | 2,847 | 0.633 |
| Parity | 0.044 | 0.066 | 0.503 | -0.085 | 0.172 | 3,368 | 0.633 |
| Delivery type | 12.407 | 8.916 | 0.164 | -5.069 | 29.882 | 3,115 | 0.633 |
| Insurance: Employee medical insurance (binary indicator) | 3.154 | 4.873 | 0.517 | -6.397 | 12.705 | 2,042 | 0.928 |
| Insurance: Resident medical insurance (binary indicator) | -0.402 | 3.486 | 0.908 | -7.234 | 6.429 | 2,215 | 0.928 |
| Insurance: Self-pay (binary indicator) | -0.247 | 2.741 | 0.928 | -5.620 | 5.126 | 2,425 | 0.928 |
| Insurance: Other insurance or assistance (binary indicator) | -1.588 | 1.711 | 0.353 | -4.942 | 1.766 | 2,821 | 0.928 |

Note: Delivery type was analyzed as a binary indicator (caesarean versus other delivery modes; source code 2 versus 0/1). Insurance category was treated as nominal: the table reports separate binary-indicator RD balance tests for employee medical insurance, resident medical insurance, self-pay, and other insurance or assistance. For binary outcomes, estimates, robust SEs, and 95% CIs are reported as risk differences in percentage points (proportion-scale values multiplied by 100). Benjamini-Hochberg q values were calculated separately for the five non-insurance covariate balance tests (age, gestational age at delivery, gravidity, parity, and delivery type) and the four insurance-indicator balance tests.

Table S7. Pre-policy placebo cutoff tests for total hospitalization cost.

| **Placebo date** | **Estimate** | **Robust SE** | **p value** | **95% CI lower** | **95% CI upper** |
| --- | --- | --- | --- | --- | --- |
| 2024-04-01 | 352.80 | 1,573.45 | 0.823 | -2,731.10 | 3,436.69 |
| 2024-07-01 | -929.17 | 1,211.53 | 0.443 | -3,303.73 | 1,445.39 |

Table S8. Bandwidth sensitivity analyses.

| **Specification** | **Estimate** | **Robust SE** | **p value** | **95% CI lower** | **95% CI upper** | **Left bandwidth** | **Right bandwidth** | **Effective sample size** |
| --- | --- | --- | --- | --- | --- | --- | --- | --- |
| Total cost bandwidth multiplier 0.5 | 270.39 | 972.78 | 0.781 | -1,636.22 | 2,177.01 | 52.089 | 52.089 | 1,394 |
| Total cost bandwidth multiplier 1 | -42.102 | 569.58 | 0.941 | -1,158.46 | 1,074.26 | 104.18 | 104.18 | 2,653 |
| Total cost bandwidth multiplier 1.5 | -65.831 | 572.73 | 0.908 | -1,188.36 | 1,056.69 | 156.27 | 156.27 | 3,909 |
| Total cost bandwidth multiplier 2 | -1.586 | 501.22 | 0.997 | -983.96 | 980.79 | 208.36 | 208.36 | 5,048 |
| Resident self-pay ratio bandwidth multiplier 0.5 | -0.431 | 5.706 | 0.940 | -11.615 | 10.754 | 60.009 | 60.009 | 334 |
| Resident self-pay ratio bandwidth multiplier 1 | -12.413 | 3.898 | 0.001 | -20.053 | -4.773 | 120.02 | 120.02 | 637 |
| Resident self-pay ratio bandwidth multiplier 1.5 | -11.586 | 3.935 | 0.003 | -19.299 | -3.874 | 180.03 | 180.03 | 934 |
| Resident self-pay ratio bandwidth multiplier 2 | -14.002 | 3.485 | <0.001 | -20.834 | -7.171 | 240.04 | 240.04 | 1,191 |
| Resident-versus-employee interaction bandwidth multiplier 0.5 | -7.094 | 5.126 | 0.166 | -17.140 | 2.952 | 46.561 | 46.561 | 1,106 |
| Resident-versus-employee interaction bandwidth multiplier 1 | -11.992 | 4.009 | 0.003 | -19.849 | -4.135 | 93.123 | 93.123 | 2,130 |
| Resident-versus-employee interaction bandwidth multiplier 1.5 | -13.490 | 3.292 | <0.001 | -19.941 | -7.038 | 139.684 | 139.684 | 3,165 |
| Resident-versus-employee interaction bandwidth multiplier 2 | -15.090 | 2.933 | <0.001 | -20.840 | -9.341 | 186.246 | 186.246 | 4,138 |

Note: Resident-insurance and resident-versus-employee interaction estimates are near zero or imprecise at 0.5 times the data-driven bandwidth but remain negative at the main and wider bandwidths.

**Table S9. Method and cohort sensitivity analyses.**

| **Specification** | **Estimate** | **Robust SE** | **p value** | **95% CI lower** | **95% CI upper** | **Left bandwidth** | **Right bandwidth** | **Effective sample size** |
| --- | --- | --- | --- | --- | --- | --- | --- | --- |
| Main eligible cohort, local linear | -42.102 | 569.58 | 0.941 | -1,158.46 | 1,074.26 | 104.18 | 104.18 | 2,653 |
| Eligible cohort, local quadratic | 221.71 | 891.05 | 0.804 | -1,524.72 | 1,968.14 | 104.18 | 104.18 | 2,653 |
| Excluding cross-cutoff stays | 186.21 | 583.89 | 0.750 | -958.19 | 1,330.60 | 100.74 | 100.74 | 2,497 |
| Including LOS >60 stays | -430.58 | 672.50 | 0.522 | -1,748.65 | 887.49 | 103.99 | 103.99 | 2,623 |
| Resident self-pay ratio, eligible cohort | -12.413 | 3.898 | 0.001 | -20.053 | -4.773 | 120.02 | 120.02 | 637 |
| Resident self-pay ratio excluding cross-cutoff stays | -17.956 | 4.597 | <0.001 | -26.965 | -8.947 | 91.645 | 91.645 | 470 |

**Table S10. Local admission-density test at the cutoff.**

| **Jackknife t statistic** | **Jackknife p value** | **Left** | **Right** | **Left sample size** | **Right sample size** |
| --- | --- | --- | --- | --- | --- |
| 2.8415278452322 | 0.004 | 98.718 | 129.29 | 4,658 | 4,032 |

## 3.2 Supplementary Figures

Supplementary Figure S1. Admission volume around the CHS-DRG 2.0 cutoff. Bars show the number of eligible admissions per 7-day bin within 180 days of 1 January 2025. The dashed line marks the admission-date cutoff. The local density test yielded a jackknife p value of 0.004. This discontinuity is interpreted as a design limitation, not as evidence that admissions were manipulated.
